# Quad-mode functional and molecular photoacoustic microscopy

**DOI:** 10.1038/s41598-018-29249-1

**Published:** 2018-07-24

**Authors:** Wei Liu, Daria M. Shcherbakova, Neel Kurupassery, Yang Li, Qifa Zhou, Vladislav V. Verkhusha, Junjie Yao

**Affiliations:** 1 Department of Biomedical Engineering, 100 Science Drive, Duke University, Durham, NC 27708 USA; 2 Department of Anatomy and Structural Biology, Albert Einstein College of Medicine, Bronx, New York USA; 3 Gruss-Lipper Biophotonics Center, Albert Einstein College of Medicine, Bronx, New York USA; 4 Department of Biomedical Engineering, University of Southern California, Los Angeles, CA 90089 USA; 5 Medicum, Faculty of Medicine, University of Helsinki, Helsinki, Finland

## Abstract

A conventional photoacoustic microscopy (PAM) system typically has to make tradeoffs between its spatial resolution and penetration depth, by choosing a fixed configuration of optical excitation and acoustic detection. The single-scale imaging capability of PAM may limit its applications in biomedical studies. Here, we report a quad-mode photoacoustic microscopy (QM-PAM) system with four complementary spatial resolutions and maximum penetration depths. For this we first developed a ring-shaped focused ultrasound transducer that has two independent elements with respective central frequencies at 20 MHz and 40 MHz, providing complementary acoustically-determined spatial resolutions and penetration depths. To accommodate the dual-element ultrasound transducer, we implemented two optical excitation modes to provide tightly- and weakly-focused light illumination. The dual-element acoustic detection combined with the two optical focusing modes can thus provide four imaging scales in a single imaging device, with consistent contrast mechanisms and co-registered field of views. We have demonstrated the multiscale morphological, functional, and molecular imaging capability of QM-PAM in the mouse head, leg and ear *in vivo*. We expect the high scale flexibility of QM-PAM will enable broad applications in preclinical studies.

## Introduction

Photoacoustic microscopy^1^ (PAM) has been proven useful in morphological^2^, functional^3^, and molecular imaging^1^ with optical absorption contrast. However, conventional PAM systems, including optical-resolution photoacoustic microscopy (OR-PAM)^4–6^ and acoustic-resolution photoacoustic microscopy (AR-PAM)^7–12^, only work at a single scale with different type of applications. OR-PAM can provide a high lateral resolution (several micrometers) and limited penetration depth (~1 mm), and thus is widely used for single cell imaging and superficial skin imaging. AR-PAM offers relatively low resolution (tens of micrometers) and deep penetration (several millimeters), and thus can be applied for deep brain imaging. Due to the complementary characteristics of OR- and AR-PAM, it is necessary to develop a PAM system that can combine OR- and AR-PAM with flexible imaging scales for different biomedical applications^13,14^. However, it is not ideal to construct the OR- and AR-PAM systems separately and combine them into a single unit, as in reference^15^, because it involves redundant system components and the time-consuming sample translation. Recently, integrated PAM systems with tunable lateral resolutions have been reported. Estrada *et al*. developed a coaxial photoacoustic imaging head by integrating a focusable optical fiber with a focused ultrasonic transducer^16^, which can provide different resolutions at the optical and acoustic focus. Xing *et al*. employed an optical fiber bundle to deliver the excitation light beam and alternated the light beam size between OR and AR excitation pattern using only one or more fiber cores^17^. Jiang *et al*. improved this concept using an electrical varifocal lens to obtain continuously tunable optically-determined lateral resolution^18^. Nevertheless, the previous methods have only focused on altering the excitation light pattern without optimizing the acoustic detection, especially for mouse brain imaging with intact skull. In this work, we propose a new quad-mode PAM (QM-PAM) system that implements different optical excitation modes and ultrasound detection frequencies to explore improved flexibilities in both spatial resolution and maximum penetration depth, for functional and molecular photoacoustic imaging^19–22^.

## Methods

### QM-PAM system

**Figure 1:**
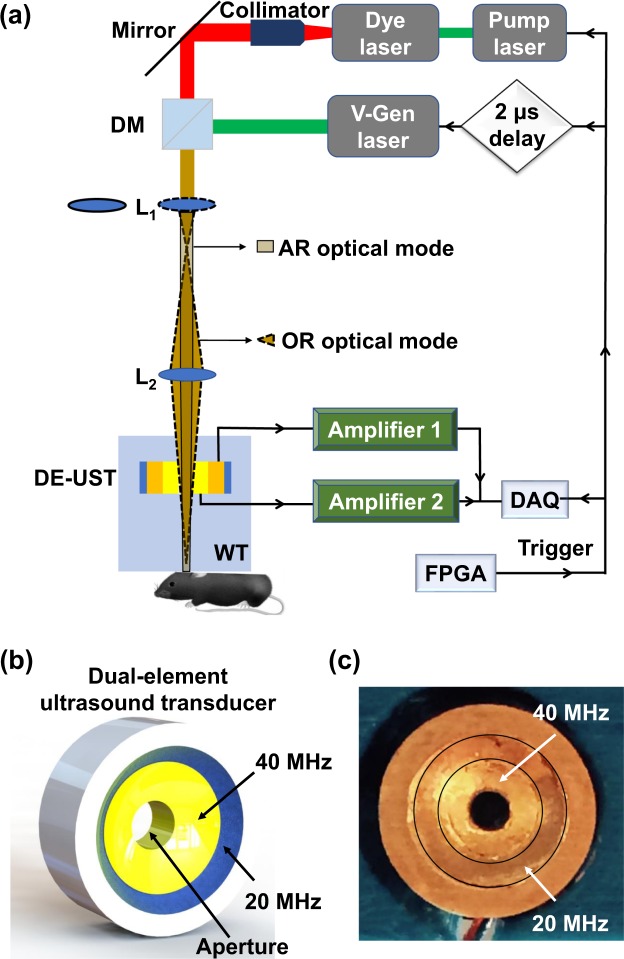
Quad-mode photoacoustic microscopy (QM-PAM). (**a**) Schematic of the QM-PAM. DAQ, data acquisition; DE-UST, dual-element ultrasound transducer; DM: dichroic mirror; L_1_ and L_2_, convex lenses; PC, personal computer; WT, water tank. The arrows show the PA data and trigger signal flow direction. (**b**) Schematic of the dual-element ultrasound transducer, showing two confocally arranged elements with respective central frequencies at 20 MHz and 40 MHz. (**c**) The photograph of the ultrasound transducer.

The schematic of the proposed QM-PAM system is shown in Fig. 1. The excitation light source is a Credo dye laser (DCM, Sirah, Grevenbroich, Germany) pumped by a pulsed laser at 532 nm (IS8II-E, EdgeWave, Würselen, Germany), with a tunable wavelength range from 620 nm to 680 nm, and an Nd: YAG fiber laser (VPFL-G-20, V-Gen, Tel Aviv, Israel) at 532 nm. The dye laser beam is firstly collimated by an objective lens (PLN4X, Olympus, Tokyo, Japan) and a convex lens with a focal length of 30 mm (AC127-030-A, Thorlabs, Newton, NJ, USA). The two laser beams are then combined by a longpass dichroic mirror (DMLP605, Thorlabs, Newton, NJ, USA). Two convex lenses, L_1_ with a focal length of 200 mm (AC127-200-A, Thorlabs, Newton, NJ, USA) and L_2_ with a focal length of 50 mm (AC127-050-A, Thorlabs, Newton, NJ, USA), are 25 cm apart to provide the tightly- and weakly-focused optical modes, respectively. L_2_ tightly focuses the collimation light beam when L_1_ is flipped out the optical pathway, providing the OR optical mode, as indicated by the solid-outlined pathway in Fig. 1(a). When L_1_ is flipped into the optical pathway, it provides the weakly-focused optical excitation, *i*.*e*., the AR optical mode, as indicated by the dashed-outlined pathway in Fig. 1(a). Therefore, the OR and AR imaging modes are alternated by switching the optical pathway. For each mode, the two lasers can be used alone or together, depending on the wavelength requirement.

The incident light passed through the central hole of a ring-shaped focused ultrasound transducer with two independent elements before reaching the surface of the imaging target. The two transducer elements have central frequencies of 20 MHz and 40 MHz, respectively, and are concentrically and confocally arranged into a single device, as shown in Fig. 1(b,c). Both transducer elements were lapped from 500-μm lithium niobate plates (Boston Piezo-Optics, Bellingham, MA, USA) to the half-wavelength thickness. The lapped material was electroplated with chrome/gold (Cr/Au) layer on both sides. Conductive backing layer was casted using conductive silver epoxy. The materials were bonded together using epoxy to form a single cylinder, and pressed by a metal ball to form the same focal length of 11.25 mm. Both transducer elements have a −6-dB bandwidth of 78%. A central aperture with a diameter of 2 mm is used to deliver light, enabling co-axial alignment of optical excitation and acoustical detection for maximum detection sensitivity. The outer diameter of the high-frequency (40 MHz) transducer element is 7.9 mm, and the outer diameter of the low-frequency (20 MHz) transducer element is 11.2 mm. The two elements have independent amplifiers with an amplification of 51 dB. The acquired PA signals of the two elements are simultaneously sampled at 500 MHz by two independent channels of a high-speed DAQ card (ATS9350, AlazarTech, Pointe-Claire, Quebec, Canada). A two-dimensional motorized stage is used for raster scanning of the sample.

### Trigger sequence

The functional and molecular imaging capabilities of QM-PAM were explored by implementing a double-wavelength illumination scheme. Both the dye laser for 640-nm light and the V-Gen laser for 532-nm light were triggered with a 2-μs delay, as shown in Fig. 1(a). The time delay between the triggers was necessary for separating the PA signals excited by the two laser pulses acquired in a single A-line. The time delay of the two laser pulses can be adjusted according to the required penetration depth.

### Imaging in mice

The *in vivo* study for demonstrating the multiscale imaging capability of the QM-PAM was conducted on the head of a female Swiss Webster mouse (8 weeks old and 21.8 g in weight) with intact scalp and skull, and on the left hind leg of the same mouse. The *in vivo* study for demonstrating the functional imaging capability of the QM-PAM was conducted on the head of a female Swiss Webster mouse (10 weeks old and 26 g in weight) with the scalp removed but skull intact. The *in vivo* study for demonstrating the molecular imaging capability of the QM-PAM was conducted on the ear of a female Swiss Webster mouse (10 weeks old and 23.1 g in weight). The protocol was approved by the Institutional Animal Care and Use Committee (IACUC) of Duke University. All methods were performed in accordance with the relevant guidelines and regulations. The hair of the mouse head, leg and ear was shaved before imaging. During the imaging, the temperature of the mouse was held at 37 °C via a heating pad and the mouse was anesthetized via isoflurane (1.5%).

### Bacteria preparation

miRFP670 expressing LMG194 (Invitrogen/ThermoFisher) *Escherichia coli* bacteria were prepared by co-transfection of the pWA23h plasmid encoding the heme oxygenase under rhamnose promoter^23^ and pBAD-miRFP670 plasmid encoding the miRFP670 near-infrared fluorescent protein under arabinose promoter^24^.

### Data availability

The datasets generated and/or analyzed during the current study are available from the corresponding author on reasonable request.

## Results and Discussions

### Resolution measurements of QM-PAM

With two optical excitation modes and the dual-element ultrasound transducer, QM-PAM can operate at four different optical-acoustic configurations: OR-HF mode (optical-resolution high-frequency), OR-LF mode (optical-resolution low-frequency), AR-HF mode (acoustic-resolution high-frequency), and AR-LF mode (acoustic-resolution low-frequency). The axial resolution is 36 µm for OR-HF mode and AR-HF mode, and 72 µm for OR-LF mode and AR-LF mode (Supplementary Fig. S1.), determined by the bandwidth of the transducer elements. For OR-HF mode and OR-LF mode, the excitation light spot is much smaller than the acoustical focus spot, and the lateral resolution is optically determined by the light spot size of 6.9 µm (see Supplementary Fig. S2). For AR-HF and AR-LF modes, the lateral resolution is determined by the acoustical focus spot size of 76.3 µm (see Supplementary Fig. S2). The 20-MHz element has a twice larger acoustic numerical aperture than the 40-MHz element. Therefore, the two elements have the same acoustical focus spot size.

### Multiscale imaging of QM-PAM *in vitro* and *in vivo*

**Figure 2:**
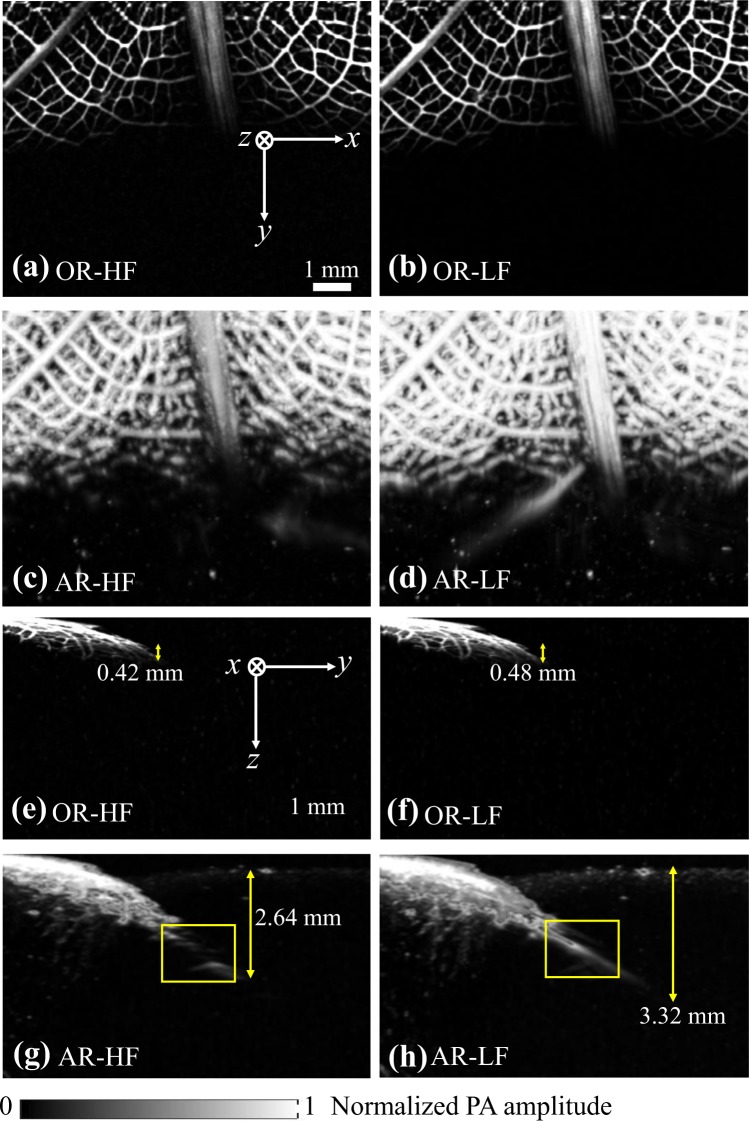
OR and AR images of the leaf skeleton phantom at four imaging modes acquired at 640-nm wavelength. (**a**–**d**) Top-view maximum projection images obtained by OR-HF, OR-LF, AR-HF and AR-LF modes. (**e**–**h**) Side-view maximum projection images obtained by OR-HF, OR-LF, AR-HF and AR-LF modes.

The multiscale imaging capability of QM-PAM was first demonstrated on a green leaf skeleton embedded in an optically scattering medium. The scattering medium was made of 1.5% (weight/volume) agar mixed with 0.2% Intralipid (reduced scattering coefficient: ~0.4 mm^−1^ at 640 nm^25^). The green leaf skeleton was obliquely inserted into the scattering medium with an inclination angle of ~45°. The image was acquired at 640 nm. The laser pulse energy was 370 nJ for OR modes and 45 μJ for AR modes. The imaging results at four different modes are shown in Fig. 2(a–d). The maximum penetration depth was quantified as the depth at which the contrast-to-noise ratio dropped to 1. As expected, the maximum penetration depths of OR and AR modes are clearly different. For OR modes, the penetration depth was around 0.4~0.5 mm (Fig. 2(e,f)) for both elements, as the tightly-focused light was strongly attenuated before reaching the optical diffusion limit (~1 mm). Therefore, the penetration depth of OR modes was optically determined. By contrast, in AR modes, the penetration depth was jointly determined by the optical and acoustical attenuation. The weakly focused light, which had >100 times stronger pulse energy than OR modes, diffused into the scattering phantom and still generated strong photoacoustic signals at depths beyond 1 mm. The maximum penetration depth was 2.64 mm for AR-HF mode and 3.32 mm for AR-LF mode (Fig. 2(g,h)). It is worth pointing out that the 20-MHz detection is less sensitive to the limited view problem that is prevalent in high-frequency PAM systems^26–28^, as marked by the yellow rectangles in Fig. 2(g,h). The improvement in detecting vertical structures is mainly due to the wider acceptance angle of the 20-MHz transducer element.

**Figure 3:**
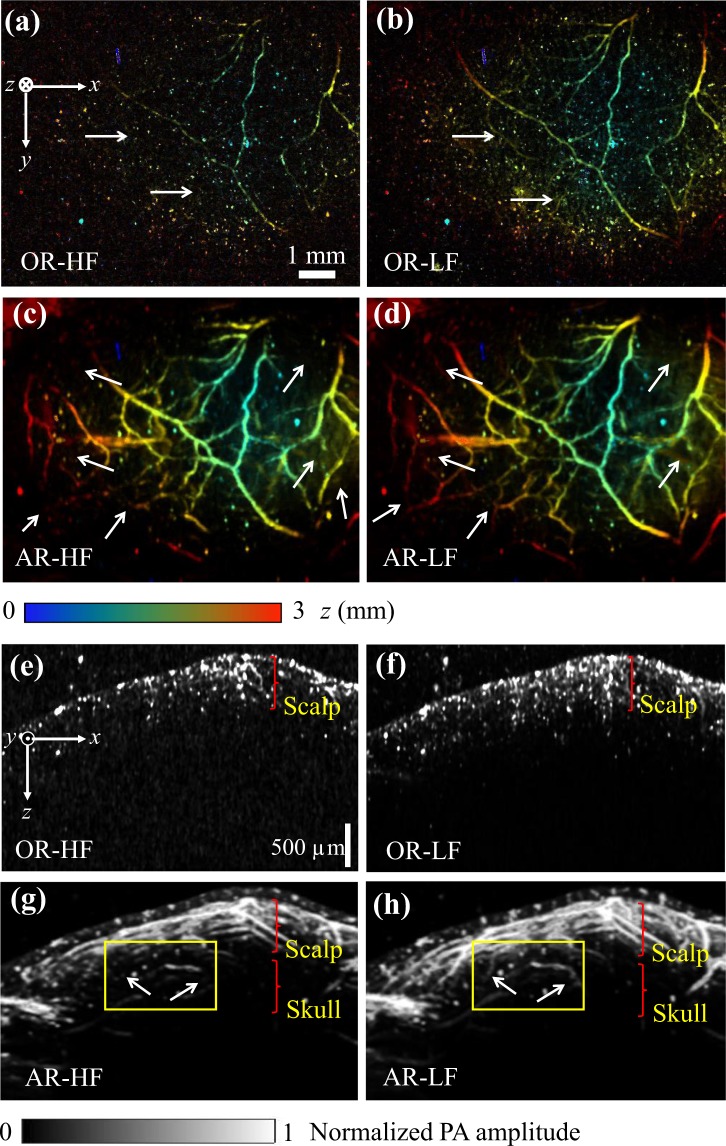
*In vivo* OR and AR images of a mouse head with intact scalp and skull, acquired at 640-nm wavelength. (**a**–**d**) Depth-encoded top-view maximum projection images of the mouse head obtained by OR and AR modes. (**e**–**h**) Side-view maximum projection images obtained by OR and AR modes.

For the mouse head imaging, the laser pulse energy at 640 nm was 370 nJ for OR modes and 45 μJ for AR modes. The head imaging results obtained at the four imaging modes are shown in Fig. 3. Blood vessels in the scalp and skull can be clearly imaged at different scales. Compared to the OR modes, the AR modes could image more blood vessels beneath the scalp, with worse resolutions. Compared to the HF modes, more vessels at larger depths were observed at LF modes due to the reduced acoustic attenuation at lower frequencies, as marked by the white arrows in Fig. 3(a–d). The side-view maximum projection images shown in Fig. 3(e–h) provide more details on penetration depths and axial resolutions. The OR modes only penetrated less than 1 mm into the scalp, while the AR modes penetrated through the scalp into the skull. Due to lower acoustic attenuation and wider detection angle, more vessels can be observed by the AR-LF mode, as marked by the yellow rectangles in Fig. 3(g,h).

**Figure 4:**
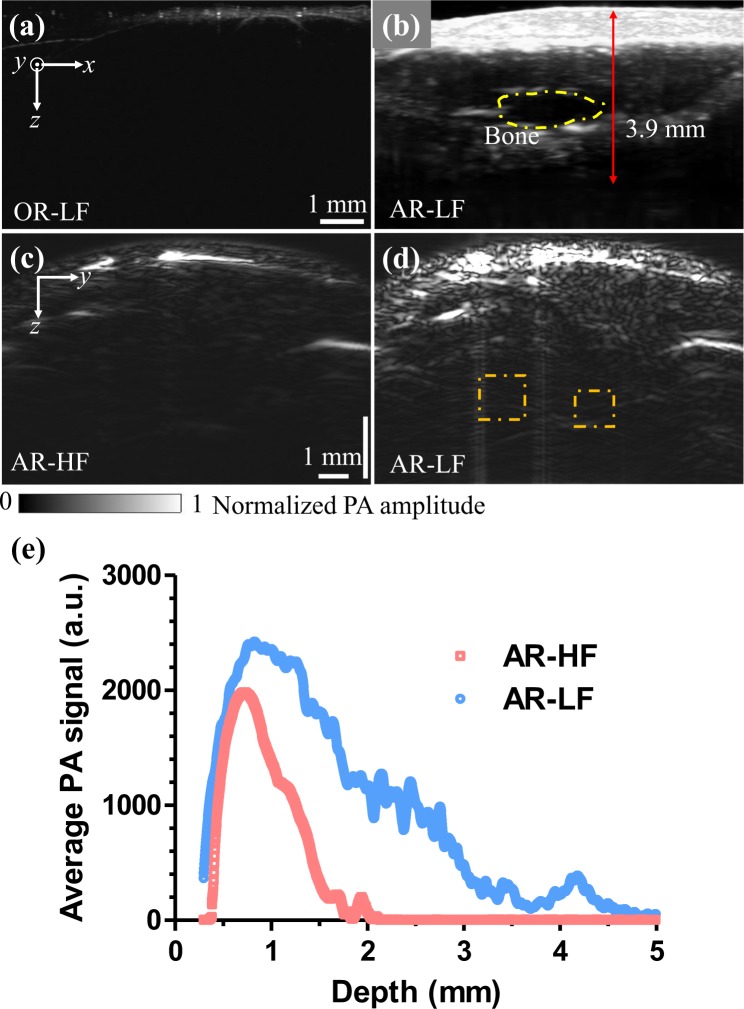
*In vivo* OR and AR images of a mouse leg acquired at 532-nm wavelength. (**a**,**b**) Side-view maximum projection images obtained by OR-LF mode and AR-LF mode. (**c**,**d**) Cross-sectional B-scan images obtained by AR-HF mode and AR-LF mode. (**e**) Average PA signal amplitudes as a function of depth by AR-HF mode and AR-LF mode.

Further, we imaged the mouse leg to demonstrate the maximum penetration depth *in vivo*. We used the 532-nm light from the EdgeWave pump laser. The laser pulse energy was 370 nJ for the OR modes and 400 μJ for the AR modes. The side-view maximum projection images of the LF modes (the larger penetration modes) are shown in Fig. 4(a,b). The penetration depth of OR-LF mode is less than 1 mm, as shown in Fig. 4(a), and the penetration depth of AR-LF mode is ~4 mm, as shown in Fig. 4(b). The outline of the leg bone can be clearly seen in the AR mode, as indicated by the dashed line in Fig. 4(b). The penetration depth of the AR mode is consistent with the previous result in the literature^29^. We have also compared the penetration difference between AR-HF and AR-LF modes, and studied the tissue’s acoustic attenuation dependence on the ultrasound frequency. To do so, we used the maximum laser pulse energy of 400 µJ to ensure adequate optical penetration. Figure 4(c,d) are the cross-sectional B-scan images of the mouse leg obtained by the two AR modes, which clearly show the penetration difference between the high-frequency and low-frequency detections. As the low-frequency detection benefited from less acoustic attenuation, deeper structures were obtained by the AR-LF mode. The dashed boxes in Fig. 4(d) show that more horizontal vessels were better imaged by AR-LF mode. Moreover, we quantified the average PA signal strength as a function of depth (Fig. 4(e)). The results have confirmed that (1) the low frequency detection had overall stronger signal strength over the entire depth, and (2) the low frequency signal had a slower decay and thus provided a deeper penetration.

Both the *in vitro* leaf phantom and the *in vivo* mouse results have demonstrated the multiscale imaging capability of QM-PAM, with progressive spatial resolutions and maximum penetration depths. It is clear that the OR modes can provide better spatial resolutions than the AR-modes, but less penetrations. Meanwhile, the 20-MHz transducer element can provide deeper penetration than the 40-MHz element, but worse axial resolutions.

### Functional imaging of QM-PAM

**Figure 5:**
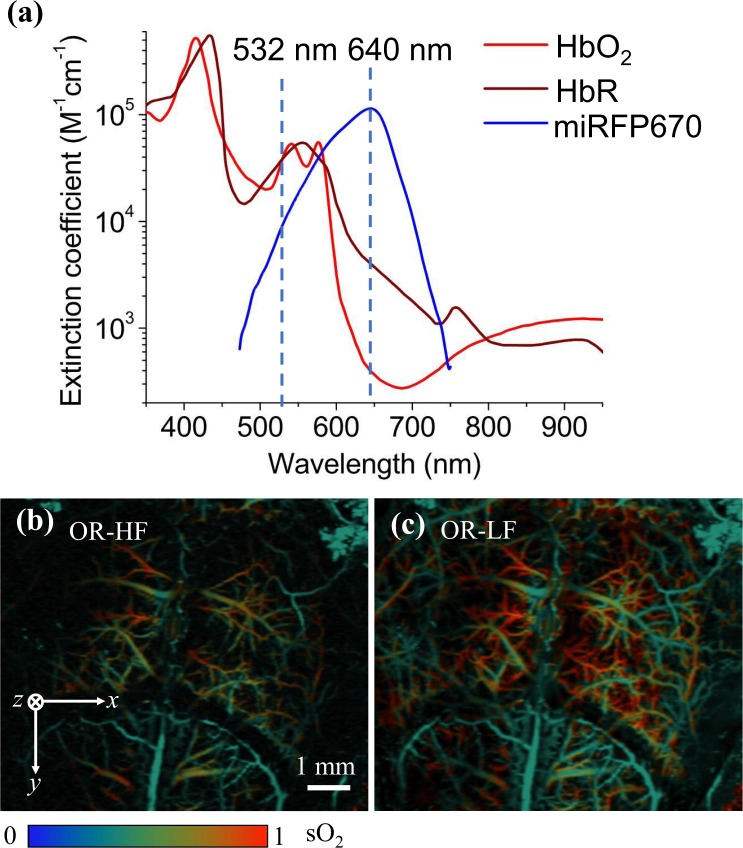
*In vivo* sO_2_ mapping of a mouse brain with the scalp removed and the skull intact. (**a**) Optical absorption spectra of the oxyhemoglobin (HbO_2_), deoxyhemoglobin (HbR) and miRFP670. (**b**,**c**) Measured sO_2_ maps of the mouse head by (**b**) OR-HF mode and (**c**) OR-LF mode with the double-wavelength illumination at 532 nm and 640 nm.

The OR modes of the QM-PAM were used to image the oxygen saturation of hemoglobin (sO_2_) in the mouse head with the scalp removed and the skull intact. A traditional linear spectral unmixing method was used to quantify the relative concentrations of oxy-hemoglobin (HbO_2_) and deoxy-hemoglobin (HbR)^30,31^, based on the different optical absorption spectra of HbO_2_ and HbR (Fig. 5(a)). Figure 5(b,c) show the measured sO_2_ of the mouse head at two OR imaging modes. In both scales, we have observed the low sO_2_ levels in the mouse skull vessels, which agrees well with our previous results^5^. We also observed that the 20-MHz transducer element provided stronger signals from the mouse cortex than the 40-MHz transduce element, reflecting the skull’s frequency-dependent acoustic attenuation. The AR modes were not used for quantifying the sO_2_, due to the unknown optical fluence at different wavelengths. The less accurate sO_2_ quantification of the AR modes is majorly due to the volumetric averaging of PA signals from unresolved small vessels and due to the wavelength-dependent optical attenuation at deeper brain regions.

### Molecular imaging of QM-PAM

The molecular imaging capability of QM-PAM was demonstrated *in vivo* on the mouse ear. The laser pulse energy was set to 100 nJ for the OR modes and 12 μJ for the AR modes. For this, the top and bottom surfaces of the ear were injected with bacteria expressing recently developed from bacterial phytochrome^32^ monomeric near-infrared fluorescent protein (FP) miRFP670 (absorbance maximum is at 641 nm and emission maximum is at 670 nm)^24^. miIRFP670 is one blue-shifted FP of the spectrally-distinct near-infrared FPs which can enable multi-spectral imaging across scales from cellular level to organismal level by labelling organs and tissues *in vivo*^32,33^. This FP has strong optical absorption at 640 nm (extinction coefficient is 87,000 M^−1^ cm^−1^) and has low absorption at 532 nm (~8,000 M^−1^ cm^−1^) whereas hemoglobin has the opposite trend, as shown in Fig. 5(a). Therefore, miRFP670 expressing bacteria can be identified by comparing the photoacoustic signals acquired at the two wavelengths.

**Figure 6:**
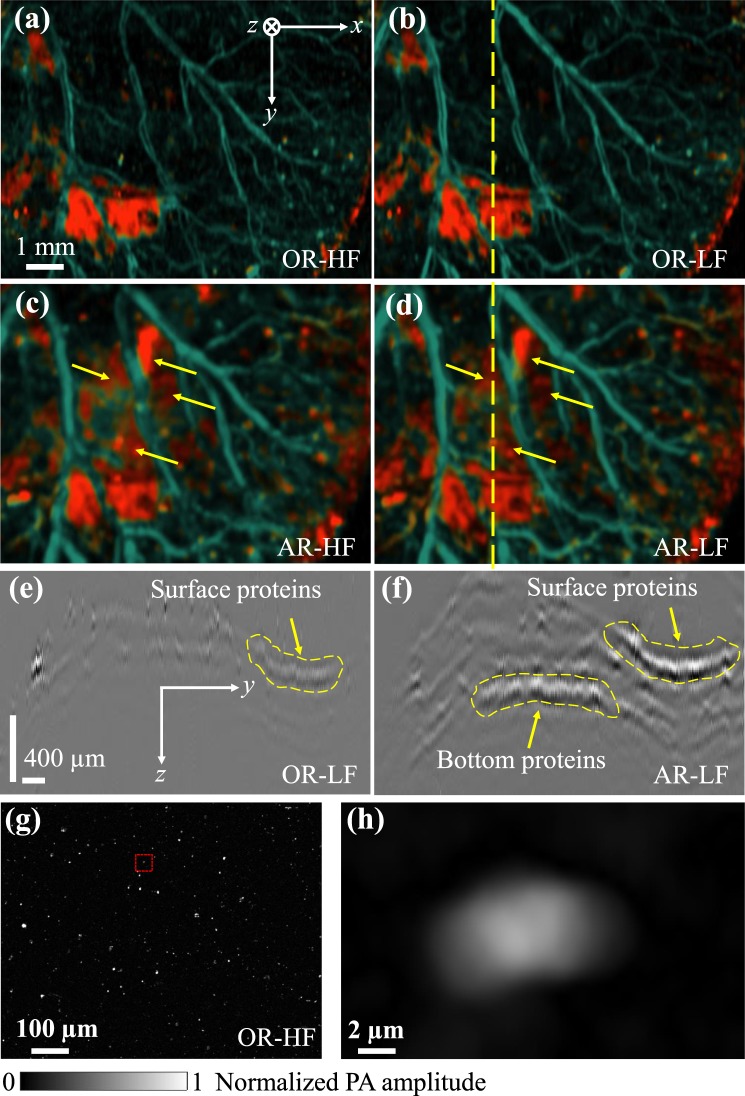
Molecular imaging of bacteria expressing miRFP670 protein. (**a**–**d**) Top-view projection images of the mouse ear injected with bacteria, obtained by OR-HF, OR-LF, AR-HF and AR-LF modes with the double-wavelength illumination at 532 nm and 640 nm. (**e**,**f**) Cross-sectional B-scan images obtained by OR-LF and AR-LF across the dashed line in (**b**,**d**). (**g**) OR-HF image of a smear of miRFP670 expressing bacteria on a glass slide, acquired at 640 nm. (**h**) Close-up image of a single bacterium as indicated by the red box in (**g**).

The imaging results at different modes are shown in Fig. 6, in which the miRFP670 signals are displayed in red and the blood signals are shown in green. While the OR images, as shown in Fig. 6(a,b), clearly show the protein distribution on the top skin surface, the AR images show the proteins on both the top and the bottom surfaces, as shown by the yellow arrows in Fig. 6(c,d). To better show the depths of the proteins, cross-sectional B-scan images of the OR-LF mode and AR-LF mode were presented along the dashed line in Figs. 6(b,d), where the positions of the top and bottom proteins have been indicated, as shown by the dashed lines in Fig. 6(e,f). To further demonstrate the high-resolution imaging capability of QM-PAM, we also imaged a smear of mIRFP670-expressing bacteria on a glass slide, using the OR-HF mode with the 640-nm wavelength illumination (Fig. 6(g)). The result clearly shows that the OR-HF mode is cable of resolving single bacteria (Fig. 6(h)).

## Conclusion

We have presented a multi-mode PAM that can provide four different length scales with complementary spatial resolutions and maximum penetration depths, as a single imaging device. The system’s function and molecular imaging capabilities have been demonstrated in the mouse head, leg and ear *in vivo*. We have shown that, by combining tightly and weakly focused optical excitation, QM-PAM can provide different lateral resolutions at the cost of penetration depths. By using a dual-element focused ultrasound transducer, QM-PAM can provide two different axial resolutions with different penetration depths. Combining the two maneuverers on light and sound, QM-PAM has achieved higher scalability than previous works.

However, one major drawback of QM-PAM is the worse acoustical lateral resolutions than that of conventional PAM systems using optical-acoustic beam combiners^34^, because the dual-element ultrasound transducer has a smaller numerical aperture. Then central aperture of the transducer also induces side-lobes in the acoustic detection. Nevertheless, the experiment results have strongly demonstrated the potential of the QM-PAM for preclinical applications, such as studying the heterogeneity of cancer hypoxic microenvironment. The acoustic resolution of QM-PAM can be improved by using ultrasound transducer with larger numerical apertures. A data fusion method that combines the acoustic signals detected by the two elements may also improve the effective detection bandwidth and reduce the side-lobes. Future studies will also include developing an image fusion algorithm that can merge images obtained at different modes.

## Electronic supplementary material

Supplementary information
