# Supplementary material for: Quad-mode functional and molecular photoacoustic microscopy
Source: Sci Rep. 2018 Jul 24;8:11123. doi: 10.1038/s41598-018-29249-1 (PMC6057954; doi:10.1038/s41598-018-29249-1)
Supplement: Supplementary file 1 — Supplementary information [file 41598_2018_29249_MOESM1_ESM.docx]

Quad-mode functional and molecular photoacoustic microscopy

Wei Liu,^1^ Daria M. Shcherbakova,^2,3^ Neel Kurupassery,^1^ Yang Li,^5^ Qifa Zhou,^4^ Vladislav V. Verkhusha^2,3,5,*^, Junjie Yao^1,*^

^1^Department of Biomedical Engineering,100 Science Drive, Duke University, Durham, NC 27708, USA

^2^Department of Anatomy and Structural Biology, Albert Einstein College of Medicine, Bronx, New York, USA

^3^Gruss-Lipper Biophotonics Center, Albert Einstein College of Medicine, Bronx, New York, USA. ^45^Department of Biomedical Engineering, University of Southern California, Los Angeles, CA 90089, USA

^5^Medicum, Faculty of Medicine, University of Helsinki, Helsinki, Finland

*Corresponding authors: [junjie.yao@duke.edu](mailto:junjie.yao@duke.edu) and vladislav.verkhusha@einstein.yu.edu

The axial resolutions of the high-frequency (HF) and low-frequency (LF) transducer elements were quantified by measuring the lateral full-width at half-maximum (FWHM) of the axial line spread function (LSF) of the PA signals, from a thin bar of the USAF test chart (58-198, Edmund Optics, Barrington, NJ, USA). Fig. S1 shows the axial resolution measurement results. The axial resolutions of the HF and LF transducer elements were 36 µm and 72 µm, respectively.

**Figure S1**


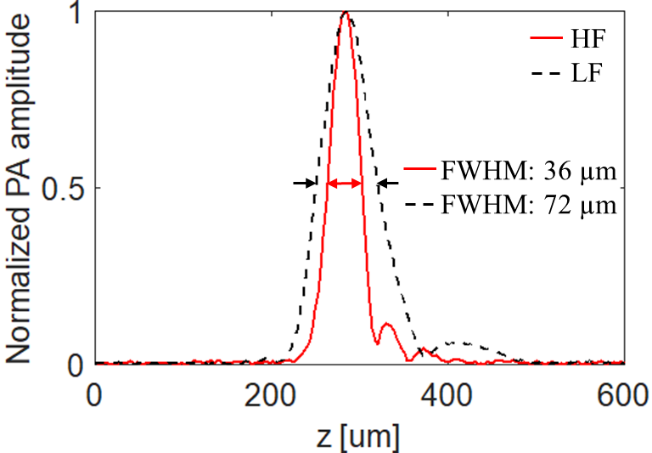


**Supplementary Fig. S1**. The axial resolution measurement results of the HF and LF transducer elements.

The lateral resolutions of the proposed QM-PAM were quantified by measuring the lateral FWHMs of the line spread functions (LSFs) of a sharp edge of the USAF test chart. The raster scanning of the sharp edge was performed with a step size of 1.25 μm for the OR modes and 5 μm for the AR modes. The measurement results for both elements under the two optical modes are shown in Fig. S2. For OR-HF and OR-LF modes, the FWHM of the LSFs were 6.9 μm, as shown in Fig. S2(a)-(d). For AR-HF and AR-LF modes, the lateral FWHMs of the LSFs were both 76.3 μm, as shown in Fig. S2(e)-(h).

**Figure S2**


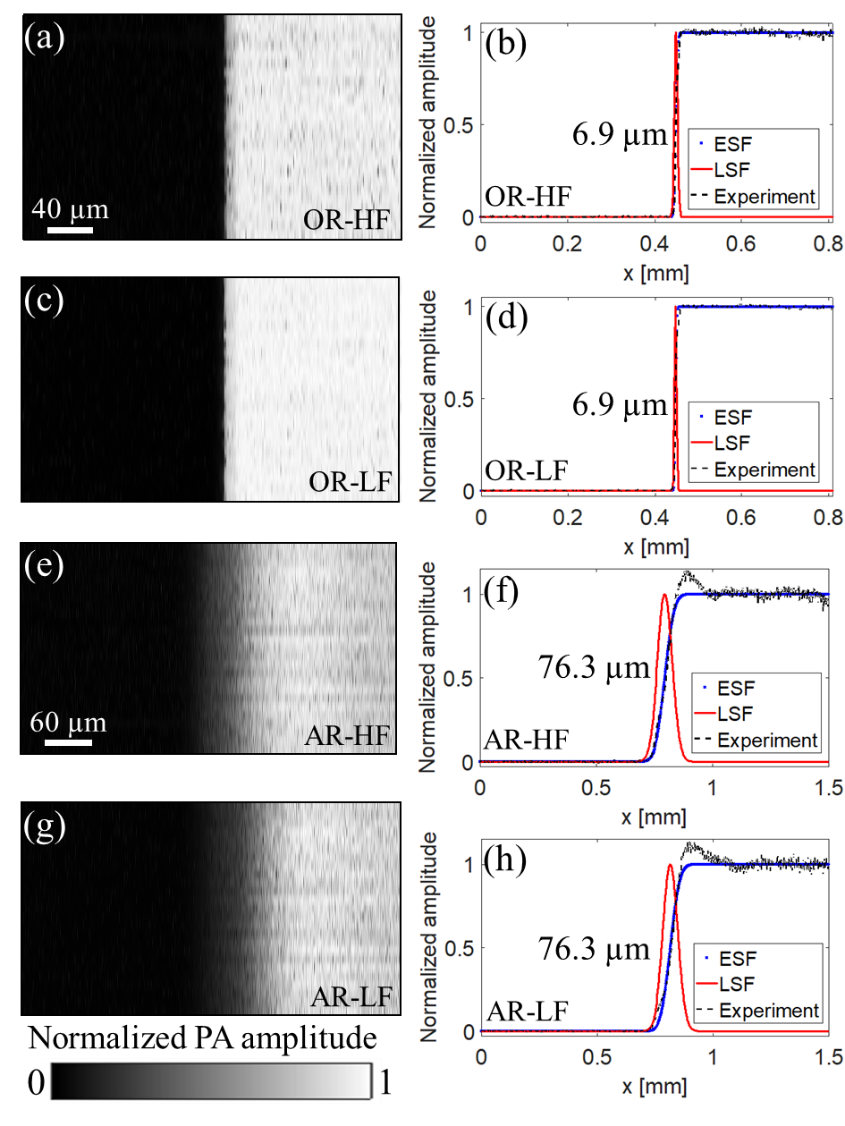


**Supplementary Fig. S2.** The lateral resolutions of four imaging modes of the QM-PAM. (a)-(d) The MAP images of the USAF chart’s sharp edge and the corresponding LSFs for the OR-LF and OR-HF modes. (e)-(h) The MAP images of the USAF chart’s sharp edge and the corresponding LSFs for the AR-LF and AR-HF modes.
